# Effects of 222 nm Far-UVC Light on Color and Lipid Oxidation in Ready-to-Eat Deli Ham and Turkey During Storage

**DOI:** 10.3390/foods15050851

**Published:** 2026-03-03

**Authors:** Jonathan J. McDonald, Yi-Cheng Wang, Bailey N. Harsh

**Affiliations:** 1 Department of Animal Sciences, University of Illinois at Urbana-Champaign, Champaign, IL 61801, USA; 2 Department of Food Science and Human Nutrition, University of Illinois at Urbana-Champaign, Champaign, IL 61801, USA; ycw@illinois.edu

**Keywords:** deli meat, far-ultraviolet C, shelf life, lipid oxidation, 222 nm

## Abstract

This study evaluated far-UVC light exposure on the visual quality of deli turkey and ham during a 5-day retail display period. Deli turkey and ham were sliced and treated with 222 nm far-UVC. Turkey received treatments: no far-UVC light (Control), low-dose 337 mJ/cm^2^ (L-UVC; Low-UVC), and high-dose 786.3 mJ/cm^2^ (H-UVC; high-UVC), while ham received Control and H-UVC treatments. Display slices were stored refrigerated and evaluated daily for color and purchase intent, with lipid oxidation measured on days 1 and 5. Data were analyzed using repeated-measure models. On day 5, H-UVC turkey slices demonstrated reduced purchase intent scores (*p* < 0.05) and a 23.7% greater total color change (ΔE; *p* < 0.01) compared with Control and L-UVC turkey. In ham, Control slices had a 9.5% greater ΔE from day 1 to 5 than H-UVC (*p* = 0.03). No differences in lipid oxidation were observed between treatments. Four hours after H-UVC treatment, deli turkey slice yellowness remained elevated compared to pre-treatment levels (*p* < 0.01). Far-UVC doses evaluated in the present study would not negatively impact the ham visual quality over a 5-day display. However, increased yellowness observed in turkey suggests that far-UVC light may be more appropriate for post-packaging surface sanitation applications than for the direct treatment of deli turkey.

## 1. Introduction

In the United States, deli department sales have increased substantially over the past decade, reflecting sustained consumer demand for ready-to-eat (RTE) meat and poultry products [1]. Although overall deli department sales have increased, the volume of meat sliced at the deli-counter declined by 11.3% from 2024 to 2025, while sales of pre-sliced, packaged deli meat have increased 5.2% over the same period [2]. As consumption of RTE deli products continues to rise, the implementation of effective food safety interventions remains critical for maintaining consumer confidence and protecting public health. Ready-to-eat deli meats are particularly vulnerable to post-lethality contamination during slicing, handling, and retail display, highlighting the need for intervention strategies that can be applied beyond the processing environment.

Ultraviolet (UV) light has been approved by the Food and Drug Administration for surface microbial control on foods and food contact materials since 2000 (21 CFR 179.39). Conventional near-UVC light (254 nm) is widely used for germicidal applications to inactive bacteria, fungi, viruses, and spores [3]. However, exposure to near-UVC light wavelengths can cause nucleic acid damage, induce apoptosis, and cause cytogenetic damage to human skin, limiting its application in food processing environments [4]. In contrast, far-UVC light (200–230 nm) has emerged as a promising alternative due to its strong antimicrobial efficacy combined with reduced penetration into mammalian tissue [5]. Previous studies have shown that far-UVC treatment can effectively reduce *Salmonella* Typhimurium and *Listeria monocytogenes* to undetectable levels in buffer, on polyethylene terephthalate film, and on stainless steel surfaces [6]. In this same study, far-UVC caused a 1.3 log reduction in *L. monocytogenes* and 1 log reduction in *S.* Typhimurium on turkey breast surfaces [6]. Additionally, far-UVC treatment has been shown to reduce fungal colony counts in cereal crops, further supporting its potential as an easily adoptable intervention technology for food systems [7]. Collectively, these findings suggest that far-UVC light may offer a practical approach for reducing microbial contamination in food handling and retail environments.

Despite its potential as a food safety intervention, any technology intended for direct application to RTE meat products must also preserve product quality. Visual appearance, particularly color, is a primary determinant of consumer purchasing decisions for deli meats [8]. Light-induced discoloration of cured and uncured meat products is often attributable to photochemical oxidation of heme pigments, including nitrosylhemochrome in cured meats, which can result in pigment dissociation and surface color fading [9]. Food safety interventions that negatively affect visual quality, even if microbiologically effective, are unlikely to be commercially viable due to consumer rejection.

Limited evidence suggests that far-UVC exposure may induce surface color changes in deli meats, such as increased yellowness following treatment [6]. However, comprehensive evaluation of far-UVC effects on visual quality, oxidative stability, and short-term retail shelf life of deli meats remains limited. In particular, the response of different deli products, such as cured ham and uncured turkey, to far-UVC exposure has not been fully characterized.

Therefore, the objective of this study was to evaluate the impact of far-UVC treatment on the visual quality and lipid oxidation of deli ham and turkey during a 5-day simulated retail display period. This work aimed to determine whether far-UVC could serve as a feasible food safety intervention without compromising key quality attributes relevant to consumer acceptance.

## 2. Materials and Methods

### 2.1. Experiment 1

#### 2.1.1. Product Procurement

Commercially available whole deli-style ham loaves (*n* = 20) and pre-sliced deli-style turkey quarter-loaves (*n* = 20) were purchased from retail grocery stores and transported to the University of Illinois Urbana-Champaign Meat Science Laboratory (Urbana, IL, USA). Products were stored under refrigerated conditions (4 °C) for no more than 4 days prior to use.

For each protein type (ham and turkey), two commercial brands were selected to represent formulation variability commonly observed at retail deli counters. Ingredient composition differed between brands; however, this variability was intentionally included to enhance the application of results to real-world retail environments. Each protein type was analyzed independently, and all statistical comparisons were made only within product type (ham or turkey) with no direct comparisons between proteins.

Ham loaves were representative of traditional restructured deli ham formulations and contained curing agents (nitrite), cure accelerators (sodium erythorbate), salt, phosphates, and antimicrobial agents (sodium diacetate). Turkey quarter-loaves were uncured (containing no nitrite or cure accelerators) and contained common restructured deli turkey ingredients such as salt, dextrose, phosphates, and sodium diacetate.

#### 2.1.2. Slicing and Sample Allocation

Ham loaves were sliced to a thickness of 1.5 cm using an automatic precision slicer (model: Automatic precision slicer SE 12 D, Bizerba USA Inc., Joppa, MD, USA). Slicing proceeded until a uniform slice diameter was obtained. From each ham loaf, five consecutive slices were collected and assigned as follows: one slice for proximate composition analysis, two slices for initial lipid oxidation, and two slices for retail display evaluation.

For turkey quarter-loaves, the outermost slice exposed to retail lighting was discarded. The subsequent slice was collected for proximate composition analysis, followed by three consecutive slices (1.2 to 1.5 cm thick) for initial lipid oxidation determination three additional consecutive slices for retail display evaluation.

#### 2.1.3. Far-UVC Treatment

Slices were assigned to control or far-UVC light (222 nm) treatment groups. Far-UVC irradiation was applied using a custom-built lighting system primarily composed of a microplasma-based far-UVC lamp (10 cm × 10 cm; Eden Park Illumination Inc., Champaign, IL, USA) housed in an acrylic frame with a lab jack on which samples could be placed at various controllable distances from the lamp.

For ham slices, treatments consisted of no far-UVC exposure (**Control**, *n* = 20) or far-UVC exposure at a target dose of 786.3 mJ/cm^2^ (**H-UVC; High-UVC**, *n* = 20). The selected dose was based on prior work demonstrating that a comparable far-UVC dose achieved approximately a 1.3 log reduction in *L. monocytogenes* on deli turkey slices [6].

For turkey slices, three treatments were applied sequentially within each loaf: Control (*n* = 20), low-dose far-UVC (Low-UVC; L-UVC; 337 mJ/cm^2^; *n* = 20). The inclusion of a lower dose was prompted by preliminary observations of visually apparent surface yellowing following high-dose treatment of turkey slices (Appendix A).

Far-UVC exposure was applied by positioning slices 7.6 cm beneath the light source. Exposure durations were 180 s (3 min) for L-UVC and 420 s (7 min) for H-UVC treatments. A 30 s pause was included midway through H-UVC exposure to minimize heat accumulation; no pause was applied during L-UVC exposure. Delivered UV dose (fluence, mJ/cm^2^) was calculated by multiplying the measured irradiation intensity values (irradiance, mW/cm^2^) by the treatment time (in seconds) [6].

#### 2.1.4. Retail Display and Storage Conditions

Immediately following treatment, slices designated for retail display were placed individually into 25.4 × 20.4 cm, 1.25 mm thick Reloc Slider deli bags (FantaPak Co., Livonia, MI, USA) labeled with a random three-digit code, and stored at 3 °C.

Ham slices were placed in cardboard boxes to prevent photooxidation-induced discoloration during storage and were held in a retail-style display cooler under LED lighting (4000K, ElectraLED, Inc., Clearwater, FL, USA) for 5 days. Packages were removed from dark storage for a 1 h period each day to conduct visual evaluations. This protocol was selected to simulate commercial handling practices, as cured deli ham is particularly susceptible to photooxidation immediately following light exposure.

In contrast, turkey slices were placed directly into simulated retail display conditions under LED lightling (4000 K) at 3 °C for 5 days. Photooxidation effects are less evident on deli turkey breast, given its pale color. This handling approach more accurately reflects typical retail practices for deli turkey.

The 5-day display period was based on manufacturer-recommended shelf-life guidelines for deli meats following package opening. Differences in post-treatment handling between ham and turkey were intentional and reflect product-specific characteristics; all statistical analyses were conducted only within each protein type.

Slices collected for proximate composition and lipid oxidation analyses were vacuum packaged and stored at −20 °C until analysis.

#### 2.1.5. Simulated Retail Evaluation

Instrumental color (CIE L*, a*, and b*) and spectral data ranging from 400 to 700 nm were collected daily during retail display with a HunterLab Miniscan EZ (Model: 4500L, Hunter Associates Laboratory Inc., Reston, VA, USA) equipped with illuminant D65, 10 degree standard observer angle, a view area of 31.8 mm and was calibrated with black and white tiles. During calibration, a deli bag was placed over the black and white tiles such that the spectrophotometer was calibrated for evaluation of slices through packaging. Readings were collected in triplicate, with the spectrophotometer applied directly to the samples surface through packaging, on the light-exposed face of each slice and reported as the average of the triplicate observations. Instrumental reflectance data were used to calculate the ratio of reflectance at 570 nm to the reflectance at 650 nm (an indicator of cured color fading where smaller values indicate less color fading; R570/650). Chroma (C*; an indicator of color saturation) was calculated with the equation C* = (a*^2^ + b*^2^)^0.5^, where larger values indicate more saturation of the primary color hue [10]. Delta E, total color change from day 1 to day 5 of retail display, was calculated with the equation ΔE = [(ΔL*)^2^ + (Δa*)^2^ + (Δb*)^2^]^0.5^ [10].

For subjective visual evaluation, six panelists evaluated ham and turkey on a 7-point hedonic scale where 1 = very definitely would not purchase, 2 = definitely would not purchase, 3 = probably would not purchase, 4 = may or may not purchase, 5 = probably would purchase, 6 = definitely would purchase, 7 = very definitely would purchase. Traditional processed meat color scales such as cured color intensity and fading were not well-suited to the objectives of this study; therefore, a willingness-to-purchase scale commonly used in consumer research [10] was applied. Trained panelists evaluated samples based on their own purchasing intent. As this was a proof-of-concept study aimed at evaluating far-UVC as a direct food safety intervention for deli meats, it was critical to assess whether any color changes were substantial enough to influence purchase decisions. Meat color strongly impacts willingness to purchase, with deviations from expected appearance known to reduce purchase intent [10].

#### 2.1.6. Proximate Composition

Slices from each meat loaf were set out to thaw at 4 °C for 24 h and then homogenized in a small food processor (model: Mini-Prep Plus, Cuisinart, Stamford, CT, USA). Moisture content was determined in duplicate using the AOAC Method 950.46 [11], and extractable lipid content was measured according to a previously described protocol [12]. Briefly, duplicate 5 g samples were weighed into dry crucibles, dried for 16–18 h in a 105 °C oven, then cooled and reweighed to determine percent moisture. Duplicate 3 g samples were weighed into dry filter paper packets, placed into a Soxhlet apparatus, extracted with a chloroform-methanol solution, dried, cooled and weighed to determine extractable lipid content. Moisture and fat were reported as a percentage of the sample weight.

#### 2.1.7. Lipid Oxidation

Lipid oxidation was evaluated with the Thiobarbituric Acid Reactive Substances (**TBARS**) method [13]. Samples of ham and turkey were thawed at 4 °C for 24 h, homogenized in a small food processor (model: Mini-Prep Plus, Cuisinart, Stamford, CT, USA). Homogenized samples (10 g for ham; 5 g for turkey) were transferred to a blender cup (Eberbach E8777.00) and combined with 1 mL 0.2 mg/mL butylated hydroxytoluene, 45.5 mL 10% trichloroacetic acid in 0.2 M of o-phosphoric acid, and either 2 mL (ham) or 1 mL (turkey) of 0.5% sulfanilamide. The mixture was blended (45 s for ham; 30 s for turkey), gravity filtered, and 0.8 mL of filtrate was transferred to two 2 mL amber microfuge tubes. One tube received 0.8 mL of deionized water (blank), while the other received 0.8 mL of 0.2 M thiobarbituric acid solution (sample). Tubes were gently inverted and incubated in dark storage at room temperature for 15–20 h. Following incubation, 150 μL from each tube was transferred to a 96-well plate for spectrophotometric analysis. Malondialdehyde (**MDA**) values were calculated from sample absorbance against a standard curve containing 0, 0.0625, 0.125, 0.25, 0.5, 0.75, 1.0, 1.5, 1.75, 1.75, and 2.0 MDA μg/mL. Samples, sample blanks and standard curves were measured for absorbance of 530 nm using a plate reader (Synergy HT Multi-Model minMicroplate Reader, BioTek Instruments Inc., Winooski, VT, USA). Values were reported as MDA μg/g of sample.

### 2.2. Experiment 2

Serial color evaluation was collected on 12 turkey slices treated with a 222 nm far-UVC light for a dose of 786.3 mJ/cm^2^ (H-UVC) on six separate days. Instrumental color (CIE L*, a*, and b*) were collected with a HunterLab Miniscan EZ equipped with illuminant D65, 10 degree standard observer angle, a view area of 31.8 mm and was calibrated with black and white tiles. Data were collected 1 min prior to far-UVC light treatment, immediately after far-UVC light treatment and 1 min, 5 min, 10 min, 15 min, 30 min, 45 min, 1 h, 2 h, and 4 h after far-UVC light treatment. This data was collected due to significant yellowing observed by researchers on turkey slice surfaces during sample treatment, yet when set evaluating samples during display approximately four hours later, the samples appeared “normal color”.

### 2.3. Statistical Analysis

Data were analyzed as a mixed model with repeated measures in SAS v9.4 (SAS Institute, Cary, NC, USA). The slice served as the experimental unit, the brand served as a random effect, the display ID served as the repeated measure, and day, far-UVC treatment, and their interaction served as fixed effects. Treatment means were sliced by retail display day. Turkey serial yellowness (b*) color evaluation data (experiment 2) were analyzed as a mixed model with repeated measures, where the slice served as the experimental unit, the slice ID served as the repeated measure, the brand and date measured served as random effects, time served as the fixed effect. For experiment 2, means were separated with the PDIFF option and compared utilizing one-sided Dunnett correction, where timepoint −1 min (one minute prior to far-UVC light treatment) served as the control that all other timepoints were compared against. Moisture and fat data were not analyzed. Assumptions of analysis of variance (ANOVA) were tested with the Levene’s test and Brown–Forsythe test for homogeneity of variances. Normality of the residuals was tested using the UNIVARIATE procedure of SAS. Significance was determined at α < 0.05.

## 3. Results

### 3.1. Experiment 1

#### 3.1.1. Ham

**Figure 1:**
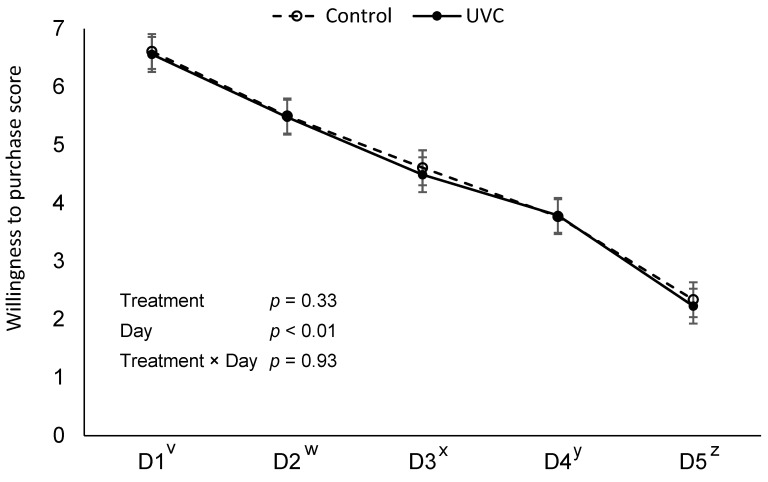
Visual willingness to purchase deli ham slices over a 5-day retail display period (D1–D5), where **Control**: no far-UVC light treatment applied and **H-UVC**: 222 nm far-UVC light treated for a dose of 786.3 mJ/cm^2^. Visual evaluation conducted on a 7-point hedonic scale where 1 = very definitely would not purchase and 7 = very definitely would purchase. ^v–z^ Letters adjacent to the day of display (*x*-axis) indicate significant differences between day marginal means (averaged across treatments), *p* < 0.05.

Willingness to purchase declined over time (*p* > 0.01); however, no treatment × day interaction was detected (*p* = 0.93, Figure 1).

**Figure 2:**
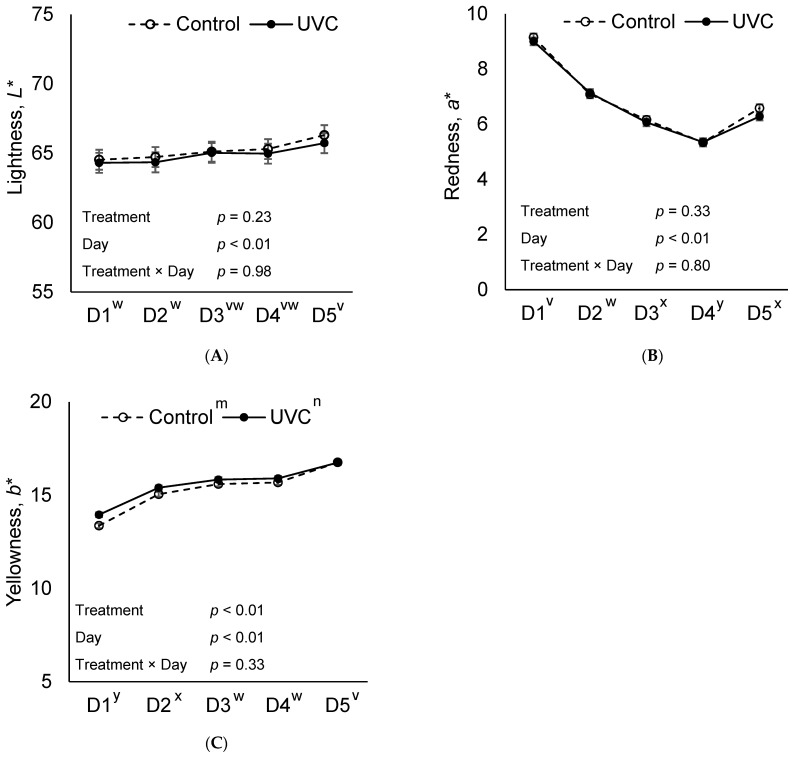
Objective lightness (L*; (**A**)), redness (a*; (**B**)), and yellowness (b*; (**C**)) of deli ham slices over a 5-day retail display period (D1–D5). Where **Control**: no far-UVC light treatment applied and **H-UVC**: 222 nm far-UVC light treated for a dose of 786.3 mJ/cm^2^. ^m,n^ Letters adjacent to treatment labels (legend) indicate significant differences between treatment marginal means (averaged across days), *p* < 0.05. ^v–y^ Letters adjacent to the day of display (*x*-axis) indicate significant differences between day marginal means (averaged across treatments), *p* < 0.05.

Instrumental color parameters L* (lightness) and a* (redness) also changed over the course of the display period, but no treatment × day interaction was observed for either parameter (*p* ≥ 0.80, Figure 2). For b* (yellowness), a treatment effect was detected (*p* < 0.01), despite no treatment × day interaction (*p* = 0.33), with H-UVC slices exhibiting 3.8% greater b* values than Control slices on day 1.

**Figure 3:**
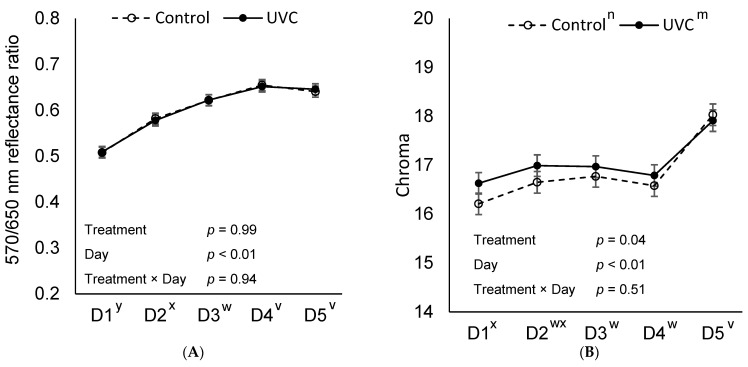
570 nm ÷ 650 nm reflectance ratio as an indicator of cured color fading (**A**), and Chroma as an indicator of color saturation (**B**) of deli ham slices over a 5-day retail display period (D1–D5), where **Control**: no far-UVC light treatment applied and **H-UVC**: 222 nm far-UVC light treated for a dose of 786.3 mJ/cm^2^. Chroma calculated by C* = (a*^2^ + b*^2^)^0.5^. ^m,n^ Letters adjacent to treatment labels (legend) indicate significant differences between treatment marginal means (averaged across days), *p* < 0.05. ^v–y^ Letters adjacent to the day of display (*x*-axis) indicate significant differences between day marginal means (averaged across treatments), *p* < 0.05.

No treatment or treatment × day interaction was observed for cured color fading ratio (R570/650; *p* ≥ 0.94; Figure 3). However, a treatment effect was present for total color change (ΔE) between days 1 and 5 (*p* < 0.03, Table 1), with Control slices displaying a 9.5% greater ΔE compared to H-UVC slices (*p* = 0.03). Additionally, a treatment effect was observed for Chroma (color intensity; *p* = 0.04) with H-UVC slices demonstrating 1.2% greater Chroma than Control slices (*p* = 0.04), likely attributable to consistent numerically higher values from day 1 through day 4.

Lipid oxidation, as measured by TBARS, did not differ between Control and H-UVC slices on either day 1 or day 5 of retail display (*p* ≥ 0.34; Table 1).

#### 3.1.2. Turkey

**Figure 4:**
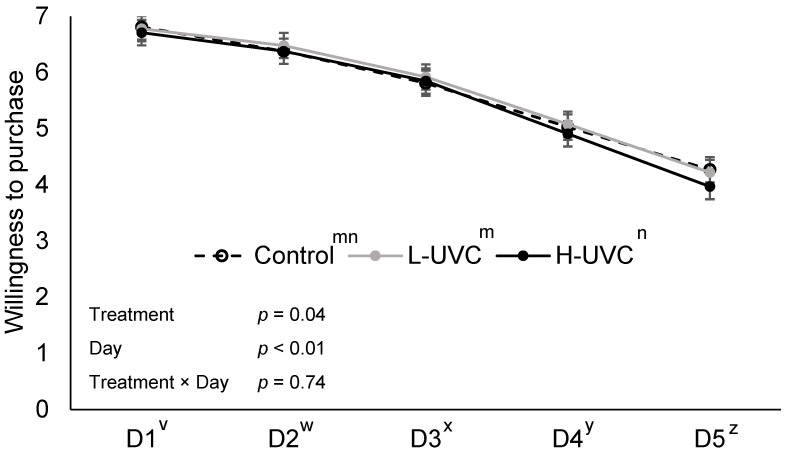
Visual willingness to purchase deli ham slices over a 5-day retail display period (D1–D5), where **Control**: No far-UVC light treatment applied; **L-UVC**: 222 nm far-UVC light treated for to a dose of 337 mJ/cm^2^; and **H-UVC**: 222 nm far-UVC light treated for to a dose of 786.3 mJ/cm^2^. Visual evaluation conducted on a 7-point hedonic scale where 1 = very definitely would not purchase and 7 = very definitely would purchase. ^m,n^ Letters adjacent to treatment labels (legend) indicate significant differences between treatment marginal means (averaged across days), *p* < 0.05. ^v–z^ Letters adjacent to the day of display (*x*-axis) indicate significant differences between day marginal means (averaged across treatments), *p* < 0.05.

Although no treatment × day interaction was observed for willingness to purchase, the main effect of treatment was present (*p* = 0.04; Figure 4).

**Figure 5:**
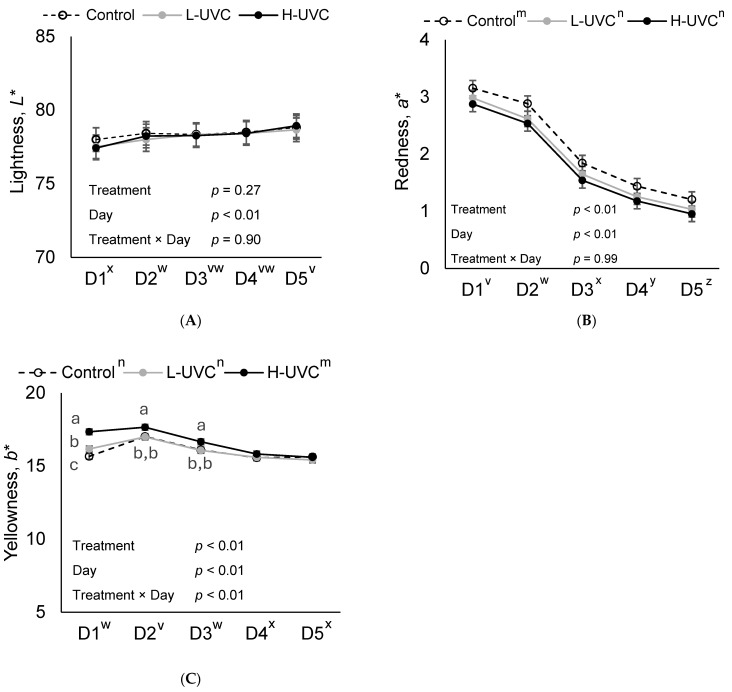
Objective lightness (L*; (**A**)), redness (a*; (**B**)), and yellowness (b*; (**C**)) of deli turkey slices over a 5-day retail display period (D1–D5), where **Control**: No far-UVC light treatment applied; **L-UVC**: 222 nm far-UVC light treated for to a dose of 337 mJ/cm^2^; **H-UVC**: 222 nm far-UVC light treated for a dose of 786.3 mJ/cm^2^. ^a–c^ Means within a panel and display day without common superscript differ, *p* < 0.05. ^m,n^ Letters adjacent to treatment labels (legend) indicate significant differences between treatment marginal means (averaged across days), *p* < 0.05. ^v–z^ Letters adjacent to the day of display (*x*-axis) indicate significant differences between day marginal means (averaged across treatments), *p* < 0.05.

This effect was likely driven by differences on day 5, where Control and L-UVC slices received higher purchase intent scores than H-UVC slices (*p* < 0.05), while no difference was observed between Control and L-UVC (*p* = 0.64). No treatment or treatment × day interaction was observed for L* (lightness; *p* ≥ 0.27; Figure 5). While no treatment × day interaction was observed for a* (redness; *p* = 0.99), there was a treatment effect for a* (redness), likely driven by difference observed on day 2 of display. On day 2, Control slices were 10.5% more red than L-UVC and H-UVC slices (*p* < 0.05), which did not differ from each other (*p* = 0.56).

**Figure 6:**
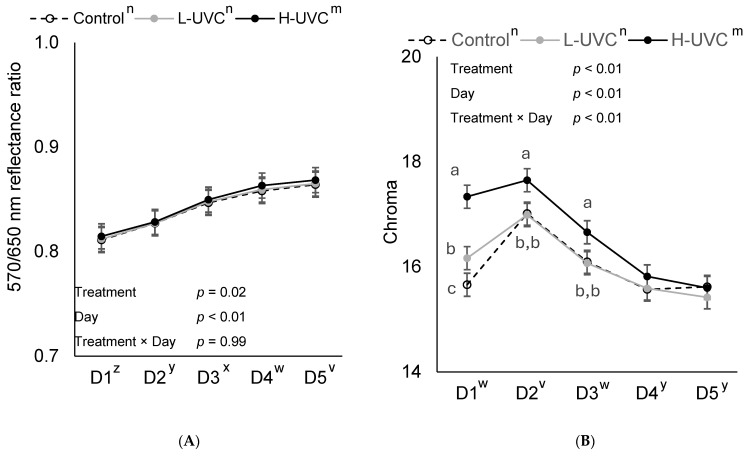
570 nm ÷ 650 nm wavelength ratio as an indicator of cured color fading (**A**), and Chroma as an indicator of color saturation (**B**) of deli turkey slices over a 5-day retail display period (D1–D5), where **Control**: No far-UVC light treatment applied; **L-UVC**: 222 nm far-UVC light treated for to a dose of 337 mJ/cm^2^; and **H-UVC**: 222 nm far-UVC light treated for a dose of 786.3 mJ/cm^2^. Chroma calculated by C* = (a*^2^ + b*^2^)^0.5^. ^a–c^ Means within a panel and display day without common superscript differ, *p* < 0.05. ^m,n^ Letters adjacent to treatment labels (legend) indicate significant differences between treatment marginal means (averaged across days), *p* < 0.05. ^v–z^ Letters adjacent to the day of display (*x*-axis) indicate significant differences between day marginal means (averaged across treatments), *p* < 0.05.

Treatment × day interactions were observed for both b* (yellowness; Figure 5) and Chroma (*p* < 0.01; Figure 6). When sliced by day, treatment effects for yellowness (b*) and Chroma (C*) were observed on display days 1, 2, and 3 (*p* < 0.02). However, no treatment effects were observed on days 4 or 5. H-UVC slices had a 7.7% more saturated color (C*) and were 8.2% more yellow on day 1 than L-UVC and Control slices (*p* < 0.05). Control slice color was 2.9% less saturated and 3.2% less yellow on day 1 of display than L-UVC slices (*p* < 0.05). On days 2 and 3 of retail display, H-UVC slice color was 3.4% and 3.3%, respectively, more saturated (C*) and 3.7% and 3.5%, respectively, and more yellow (*p* < 0.01) than L-UVC and Control slices which did not differ from each other (*p* > 0.75).

No treatment × day interaction was observed for cured color fading ratio (R570/650; *p* = 0.99; Figure 6). However, there was a treatment effect for ΔE (total color change) and cured color fading ratio (R570/650) from day 1 to day 5 of display (*p* ≤ 0.02). H-UVC slices had, on average, a 23.7% greater color change (ΔE) than L-UVC and Control (*p* < 0.01) slices which did not differ from each other (*p* = 0.08). Control and H-UVC cured color fading ratio did not differ (*p* = 0.62) and was greater than L-UVC turkey slices (*p* ≤ 0.03).

Lipid oxidation, as measured by TBARS, did not differ between treatments at either day 1 or day 5 of retail display (*p* ≥ 0.51; Table 1).

### 3.2. Experiment 2

**Figure 7:**
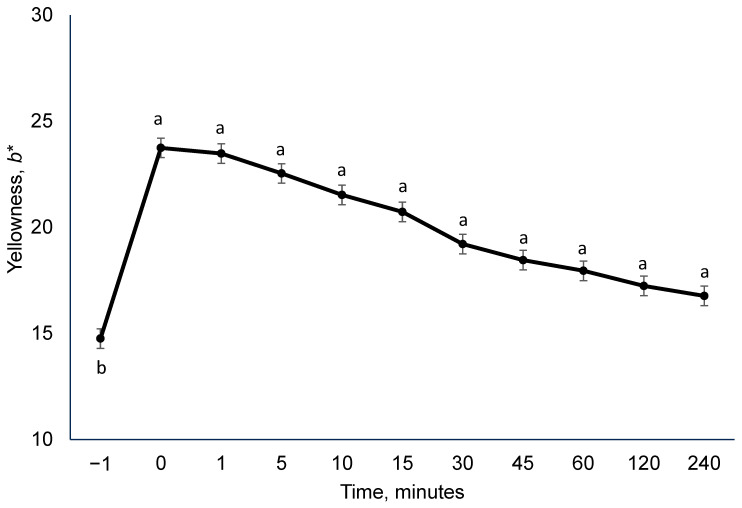
Serial evaluation of surface yellowness (b*) of deli turkey slices following high-dose far-UVC treatment. Measurements were collected from 1 min prior to treatment (pre-treatment baseline; −1 min) through 240 min post-treatment. Turkey slices were treated with 222 nm far-UVC light at a dose of 786.3 mJ/cm^2^ (H-UVC). Statistical analysis was performed using Dunnett’s multiple comparisons test, with each post-treatment time point compared directly to the pre-treatment baseline. ^a,b^ Means without a common superscript differ from the pre-treatment value (−1 min), *p* < 0.05.

The effect of time was observed for yellowness (b*) during serial color evaluation (*p* < 0.01; Figure 7). Deli turkey slices at one minute prior to far-UVC light (−1, control) were less yellow than all other time points (*p* < 0.01). Even at four hours post far-UVC light treatment, the yellowness of deli turkey slices did not return to pre-treatment levels (*p* < 0.01; Table 2).

## 4. Discussion

### 4.1. Experiment 1

This study was intentionally designed as a proof-of-concept evaluation of far-UVC as a direct food safety intervention for deli meats, with primary emphasis on visual quality and lipid oxidation due to their established relevance to consumer acceptance and shelf life. While other functional and physicochemical attributes, such as protein oxidation, water-holding capacity, or textural changes, may also be influenced by photochemical treatments, the inclusion of these measures was beyond the scope of the present investigation.

#### 4.1.1. Ham

On day 1, H-UVC ham slices were 3.85% more yellow than control ham slices. Although this difference was statically significant, the 0.6-unit increase in b* is unlikely to be visually perceptible, as supported by visual panel scores. Moreover, the 0.41 ΔE difference from control ham to H-UVC would not be perceptible either [13]. Delta E values less than or equal to 1 would not be perceptible by human eyes, values between 1 and 2 would be perceptible through close observation, values 2 through 10 would be perceptible at a glance, 11 through 49 are colors are more similar than opposite and a value of 100 indicates colors are exact opposites [13,14]. Cured color fading (570/650 nm ratio) was not affected by far-UVC light treatment, contrary to the authors’ initial expectation that light exposure would induce fading. Consumers rely heavily on cured color as a visual quality indicator, with differences in cured color attributes playing a key role in product evaluation and purchase-related decisions [15]. Given that retail case lighting alone can cause ham cured color fading, it was notable that far-UVC treatment did not exacerbate color fading over five days of retail display.

Lipid oxidation is a key determinant in a product’s shelf life and can be affected by the total amount of fat present [16]. Deli ham meat in the present study had, on average, 71.73% moisture and 5.51% fat which did not deviate from the previously reported literature [17,18], and would not be expected to cause lipid oxidation at a greater rate. Ham MDA values were below 0.4 μg/g on day 1 and 0.6 μg/g on day 5 with no difference observed between treatments, which indicated far-UVC had no effect on lipid oxidation in deli ham. Based off previously reported sensory data, MDA values reported in the present study indicate little or no off-flavor and off-odor would be detected on day 1 and a low chance of off-flavor and off-odor detected on day 5, since day 1 values were below 0.5 μg/g and day 5 were below 1.0 μg/g [19,20]. The visual quality of deli ham was largely unaffected by far-UVC light treatment. Given the lack of effect on lipid oxidation, it is unlikely that the use of far-UVC light on deli ham would result in an increased presence of off-flavor development; however, eating characteristics such as juiciness, texture profile, and flavor were not investigated in the present study.

#### 4.1.2. Turkey

Although differences in yellowness and color saturation were observed between H-UVC and control turkey slices on days 1 through 3, the magnitude of these differences diminished over time and was no longer present by day 4, with no associated changes in lightness or redness. Considering this, the USDA recommends lunch meat that is deli sliced or store-prepared to be consumed or stored three to five days under refrigeration—the window in which objective color differences were observed in this study [21]. These differences could cause consumers to reject the product; however, differences observed on days 1, 2, and 3 for yellowness did not exceed 2 b* units, which would not be expected to be perceptible as indicated by visual purchasing intent scores. Additionally, far-UVC-treated deli turkey had a greater color saturation on day 1, where H-UVC turkey color saturation was greater through the first three days of display. This observation was expected, however, as b* values are used in C* calculation. Greater color saturation, or vividness, could be seen as a positive effect of far-UVC light treatment, yet color saturation differences reported were not enough to be observable through subjective visual evaluation. Interestingly, there was a treatment effect found on day 5 of display, where H-UVC turkey had a 6.48% less chance of being purchased than Control and L-UVC turkey. This may indicate that deli turkey treated with a high dose of far-UVC light could fail visual inspection at a greater rate if held past 5 days on retail shelves.

Lower purchasing intent scores observed on day 5 were likely not attributable to greater lipid oxidation as MDA values were not different between treatments on day 5. Deli turkey meat in the present study contained 73.62% moisture and 1.86% fat. However, because turkey meat typically contains a greater proportion of unsaturated, membrane-bound lipids and low concentrations of endogenous antioxidants, comparatively high MDA values were expected despite the low total fat content [22]. While day 1 MDA averaged 3.53 μg/g meat and day 5 MDA averaged 7.78 μg/g, these values do not deviate from reported cooked turkey values and would not be expected to cause any differences in flavor [23,24]. Moreover, since far-UVC light did not impact the extent of lipid oxidation in deli turkey meat, the differences observed in visual purchasing intent on day are not likely attributable to lipid oxidation.

A possible explanation for the change in purchasing intent on day 5, elaborated later in this paper, could be increased protein oxidation; however, crude protein or free amino acid abundance were not quantified in the present study. Far-UVC light treatment doses used in the present study did not have a detrimental effect on visual quality during a 5-day retail display period; however, the sudden color change immediately post-treatment raises concerns for use at retail deli counters. The results suggest that far-UVC light treatment may not be practical for immediate use on fresh sliced deli meat in brick-and-mortar stores due to immediate color changes and long treatment times; however, far-UVC light treatment may have applications in large production plants as a post-packing microbial intervention, where immediate color change is not a concern. It may also be used for deli counters and slicing equipment for other types of deli meat products that are not susceptible to color changes (such as ham). Although the treatment times used in the experiments above (i.e., 7 min) may be considered long in some scenarios, such as at deli counters, advances in technology and equipment may reduce these durations; long treatment times used in this study may be reduced, opening far-UVC light treatment to other applications in the meat industry.

### 4.2. Experiment 2

In experiment 1, an intense yellow color was initially observed following treatment; however, the discoloration had nearly disappeared by the time samples were prepared for retail display and subsequent instrumental and visual color evaluation. This unexpected change prompted further investigation and served as the basis for Experiment 2. An 8.98 b* unit increase from pre-light treatment to immediately post-treatment was observed. Moreover, the minimum pre-treatment yellowness value, 14.24, nearly doubled when compared to the maximum yellowness value, 26.88, immediately after treatment. Further, immediately after far-UVC light treatment, the observed range between minimum and maximum yellowness (b*) values began as wide and then started to become narrower as time after far-UVC light treatment passed. Specifically, absolute yellowness (b*) range of 7.5 units at 1 min, 3.4 units at 30 min, and 2.6 units at 120 min post-treatment. These observations may suggest incomplete light surface penetration, pockets of reactive substances, or compound degradation. Minimal lipid oxidation products reported in Exp. 1, on day 1 post-light-treatment, indicate the severe yellowing observed in turkey deli meat far-UVC light was not likely to have been caused by lipid oxidation.

In pharmaceutical formulations, Histidine (**His**)-based buffers are recognized as being prone to photo-oxidation, with patented stabilization methods developed to prevent solution yellowing [25]. Histidine’s imidazole group has been demonstrated to be susceptible to photo-oxidation and is a known chromophore when exposed to heat energy [26]. Far-UVC light specifically enhances oxygen reactivity with the His imidazole group and can develop a yellow color during accelerated light exposure and degradation [27,28]. Moreover, UVC-induced oxidation of His produced Aspartic Acid (**Asp**) and Asparagine (**Asn**) almost exclusively [28]. Investigating the amounts of Asp and Asn pre- and post-far-UVC light treatment could help determine the mechanism behind the yellowing. When comparing the abundance of free amino acids in turkey and pork (i.e., meats used for making ham), turkey had three times more total free amino acids and 13 times more His than pork [29,30]. Additionally, His excitation was at a wavelength of 220 nm, with its fluorescence observed at 360 nm [31]. The spectrophotometer used in the present study estimates reflectance from transmitted light; therefore, far-UVC exposure likely modified the slice’s surface chemistry or optical behavior, lowering transmittance. Two explanations are plausible. First, far-UVC may have induced fluorescence, causing the slice to emit light, reduce measurable reflectance, and contribute to the yellow appearance. Second, far-UVC exposure may have changed the surface microstructure which would have promoted light scattering resulting in decreased overall reflectance and possibly contributed to the yellow appearance. Therefore, our hypothesis is that the free His located on the surface or near the surface of H-UVC-treated turkey deli meat was photo-oxidized, degraded, or a combination thereof, resulting in the yellow color. Future research to investigate this hypothesis should focus on capturing the 360 nm wavelength, and determination of Asp and Asn pre- and post-far-UVC light treatment.

## 5. Conclusions

In both ham and turkey deli meat products, minimal objective color differences were found between far-UVC-light-treated and non-treated deli meat with reported differences not affecting willingness to purchase scores for evaluators. Limitations of the far-UVC technology are long treatment times and intense surface yellowing immediately post-treatment in the turkey deli samples, which would likely result in consumer rejection. However, the long treatment time could be addressed as technology and equipment advance, for example by delivering higher power over a shorter period. Overall, the far-UVC light treatment doses used in the present study would not be expected to detrimentally affect meat quality over a 5-day shelf display. Future research should investigate far-UVC lights effect on sensory characteristics, efficacy through film packages, efficacy for slicer equipment, and potentially identify the cause of turkey surface yellowing.

## Figures and Tables

**Table 1:** Total color change (ΔE) and initial and final lipid oxidation of deli ham and deli turkey meat subjected to 5 days of retail display.

	Deli Meat Type ^1^								
	Ham				Turkey				
	Control ^2^	H-UVC ^3^	SEM	*p* value	Control	L-UVC ^4^	H-UVC	SEM	*p* value
Day 1 to 5 ΔE ^5^	4.85 ^a^	4.44 ^b^	0.13	0.03	2.21 ^b^	2.47 ^a,b^	3.07 ^a^	0.19	<0.01
MDA ^6^, µg/g meat									
Day 1	0.30	0.57	0.04	0.34	3.53	3.59	3.48	0.28	0.96
Day 5	0.36	0.62	0.04	0.41	8.16	7.56	7.64	0.28	0.51

^1^ Protein source of deli meat loaves. ^2^ Control: no far-UVC light treatment applied. ^3^ H-UVC: 222 nm far-UVC light treated for a dose of 786.3 mJ/cm^2^. ^4^ L-UVC: 222 nm far-UVC light treated for a dose of 337 mJ/cm^2^. ^5^ ΔE = [(ΔL*)^2^ + (Δa*)^2^ + (Δb*)^2^]^0.5^. ^6^ Malondialdehyde. ^a,b^ Means within protein source and row without common superscript, differ *p* < 0.05.

**Table 2:** Serial yellowness (b*) summary statistics of deli turkey meat treated with a far-UVC light ^1^.

Time ^2^, min	Mean	Minimum	Maximum	SEM
−1	14.76	14.24	15.90	0.37
0	23.74	19.81	26.88	0.91
1	23.47	19.16	26.62	0.82
5	22.54	19.27	25.13	0.62
10	21.53	18.57	23.99	0.58
15	20.72	17.73	22.62	0.52
30	19.21	17.35	20.79	0.45
45	18.45	16.96	19.77	0.42
60	17.95	16.53	19.01	0.40
120	17.24	15.91	18.53	0.38
240	16.77	15.87	17.88	0.36

^1^ 222 nm far-UVC lamp for a dose of 786.3 mJ/cm^2^, ^2^ where −1 min represents collection time 1 min prior to far-UVC light exposure and 0 min represents collection time immediately following far-UVC light exposure.

**Figure A1:**
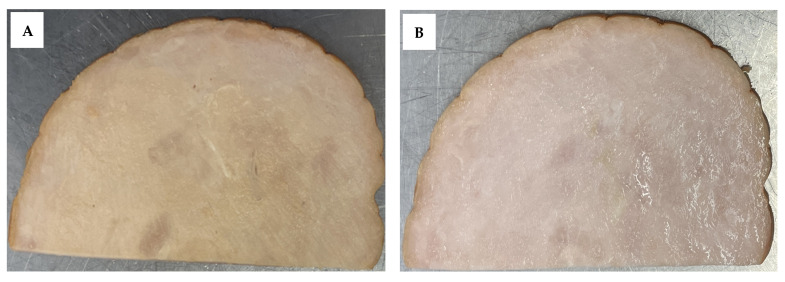
(**A**) H-UVC deli turkey slice face photograph captured after far-UVC light treatment. (**B**) Non-treated slice face of an H-UVC treated deli turkey slice captured after far-UVC light treatment.

## Data Availability

The original contributions presented in the study are included in the article, further inquiries can be directed to the corresponding author.
